# Deep-learning-based cross-modality translation from Stokes image to bright-field contrast

**DOI:** 10.1117/1.JBO.28.10.102911

**Published:** 2023-10-20

**Authors:** Shilong Wei, Lu Si, Tongyu Huang, Shan Du, Yue Yao, Yang Dong, Hui Ma

**Affiliations:** a Tsinghua University, Shenzhen International Graduate School, Shenzhen, China; b Tsinghua University, Department of Biomedical Engineering, Beijing, China; c University of Chinese Academy of Sciences, Shenzhen Hospital, Department of Pathology, Shenzhen, China; d Tsinghua University, Department of Physics, Beijing, China

**Keywords:** cross-modality, snapshot Stokes images, cycle generative adversarial network, polarization

## Abstract

**Significance:**

Mueller matrix (MM) microscopy has proven to be a powerful tool for probing microstructural characteristics of biological samples down to subwavelength scale. However, in clinical practice, doctors usually rely on bright-field microscopy images of stained tissue slides to identify characteristic features of specific diseases and make accurate diagnosis. Cross-modality translation based on polarization imaging helps to improve the efficiency and stability in analyzing sample properties from different modalities for pathologists.

**Aim:**

In this work, we propose a computational image translation technique based on deep learning to enable bright-field microscopy contrast using snapshot Stokes images of stained pathological tissue slides. Taking Stokes images as input instead of MM images allows the translated bright-field images to be unaffected by variations of light source and samples.

**Approach:**

We adopted CycleGAN as the translation model to avoid requirements on co-registered image pairs in the training. This method can generate images that are equivalent to the bright-field images with different staining styles on the same region.

**Results:**

Pathological slices of liver and breast tissues with hematoxylin and eosin staining and lung tissues with two types of immunohistochemistry staining, i.e., thyroid transcription factor-1 and Ki-67, were used to demonstrate the effectiveness of our method. The output results were evaluated by four image quality assessment methods.

**Conclusions:**

By comparing the cross-modality translation performance with MM images, we found that the Stokes images, with the advantages of faster acquisition and independence from light intensity and image registration, can be well translated to bright-field images.

## 1 Introduction

Polarimetric imaging, which can probe abundant microstructural information of tissues, is attracting increasing attention and interest in the biomedical field.^1^^–^^4^ The polarization state of light can be described by a four-component Stokes vector $S={\left(S_{0},S_{1},S_{2},S_{3}\right)}^{⊺}$. The Mueller matrix (MM) describes the ability of a medium to convert the incident polarization state $S_{\text{in}}$ into $S_{\text{out}}$ when the light propagating and scattering in it, which can be formalized as $S_{\text{out}}=MS_{\text{in}}$.^5^ MM provides a comprehensive description about the polarization properties of samples, and many polarimetric parameters (e.g., depolarization, retardance, and diattenuation) extracted from this 4×4 matrix are closely related to some microstructures. Polarimetric techniques have assisted the diagnosis of abnormal or cancerous lesions both *in vivo* and *ex vivo*, e.g., brain,^6^ esophagus,^7^ cervix,^8^ liver,^9^ breast,^10^ and gastric^11^ tissues.

In biomedical and clinical scenarios, different modalities are usually required to highlight and analyze different components in the same sample based on their respective strengths. Pathologists need to access them in different ways, which may require preparing multiple imaging systems or changing hardware. Cross-modality translation techniques can blend microscopy and computation to transform images between microscopic imaging systems.^12^ Deep learning, which is able to learn abstract feature representations in a hierarchical way and discover hidden data structures,^13^ has proven to be a powerful tool for various inference tasks in the field of microscopic image analysis.^14^^–^^17^ Considering the complex patterns and dependences contained in different high-dimensional microscopic modality data, deep learning approaches are mainly adopted in cross-modality translation works. There are many deep-learning-based methods being demonstrated for transformations between different imaging modalities, e.g., from total internal reflection fluorescence (TIRF) microscopy images into TIRF-based structured illumination microscopy (TIRF-SIM) equivalent images,^18^^,^^19^ from diffraction-limited confocal microscopy images into stimulated emission depletion microscopy equivalent images^18^ and from wide-field fluorescence microscopy images into optically sectioned SIM images.^20^

Bright-field microscopy is often considered the gold standard in histological analysis. It is often combined with other microscopic modalities to probe the sample from different levels. Some previous studies have reached the transformation to bright-field contrast from other microscopic modalities, e.g., holographic microscopy.^21^^,^^22^ Mueller matrix microscopy (MMM) and bright-field microscopy contain different contrast information. They have different imaging principles, and each has its advantages. In previous studies, we proposed a cross-modality transformation from MM microscopy images to bright-field microscopy images^23^ based on a conditional generative adversarial network (cGAN)^24^ without changing the optical path design. However, to obtain a MM image, four exposures of the dual division of the focal plane (DoFP) polarimeter-based system^25^ are required, which will be affected by light intensity fluctuations and co-registration of polarimetric images for imaging quality.^26^ Meanwhile, the acquisition process is lengthy compared to obtaining a snapshot Stokes polarimetric image. In this work, we adopt Stokes images as input in the cross-modality transformation to bright-field microscopy. This method can output a corresponding virtual bright-field equivalent from a Stokes image, which combines both the snapshot imaging of MM microscopy and the high contrast of bright-field microscopy. In this case, we refer to this approach as “bright-field snapshot MM microscopy.”

In addition to only simply transforming between different microscopic modalities, deep learning-based cross-modality microscopic translation can also algorithmically create a physical transformation on a sample, e.g., for virtual staining of label-free tissue samples.^12^ There are different kinds of staining styles, each of which can express different contrast information. The process of traditional chemical staining is time-consuming, laborious, and may contain toxic chemical reagents.^27^^–^^29^ Computational staining, a data post-processing method, can generate various staining results without using real chemical reagents.^30^^–^^32^ It has been proven that autofluorescence,^33^^–^^35^ phase,^36^ bright-field,^37^ and total-absorption photoacoustic remote sensing images^38^ of a label-free tissue sample can be virtually performed to hematoxylin and eosin (H&E) and/or other staining domains by a deep neural network. Realistic-looking H&E images can be also generated from immunofluorescence images stained for DAPI and ribosomal S6.^39^ Usually, training a deep model requires the input and the ground truth image to be well co-registered at the pixel-level (e.g., cGAN), which requires meticulously capturing images of the sample and is very painstaking in the pre-processing of the data. Each immunohistochemistry (IHC) staining is usually costly and the destructive histochemical staining procedures are irreversible. This makes it hard or sometimes impossible to obtain multiple staining on the same tissue section. In this work, we capture images of H&E staining samples using a polarimetric imaging approach and build a deep-learning model to translate them to bright-field microscopy images of unpaired tissue slides in the training phase. To visually compare the transformation performance, we used adjacent tissue sections, which share approximately the same contour and structural characteristics, as the ground truth.

In summary, we measured already-existing tissue slides for model training and no other preliminaries are required. We adopted deep learning from the point-of-data-driven method, statistical mapping between image domains of snapshot MM microscopy, and bright-field microscopy aiming at the cross-modality microscopic translation. Our contributions can be summarized as follows.

• We propose a “bright-field snapshot MM microscopy” method that transforms Stokes images obtained by MM microscopy to bright-field microscopy images. It is demonstrated on paired images of H&E stained breast and liver tissues. This method is not sensitive to the incident polarization states of light and resolutions of polarimetric images, which makes the system more robust and practical.
• We demonstrate that the transformation performance using Stokes images as input is close to the results of MM images. The Stokes polarimetry is unaffected by system and sample instabilities and allows faster image acquisition compared to MM polarimetry, so it is superior for improving the system stability and transformation efficiency in MM microscopy.
• We employ the cycle generative adversarial network (CycleGAN)^40^ to transform between the two domains without preparing paired images, which can greatly improve the efficiency of the data pre-processing procedures and avoid possible errors due to image registration. This method is necessary when performing IHC virtual staining on H&E stained lung tissues as there is no ground truth corresponding to the staining style images in the same tissue region.

We organize the sections in this paper as follows. Section 2 introduces the experimental setup, sample preparation and data processing. Section 3 shows architecture and principle of the deep learning model CycleGAN. Section 4 gives cross-modality translation results on H&E and IHC stained tissues, respectively, and Sec. 5 provides discussion and conclusion.

## 2 Materials and Methods

### 2.1 Stokes Polarimetry and Mueller Matrix Polarimetry

**Fig. 1:**
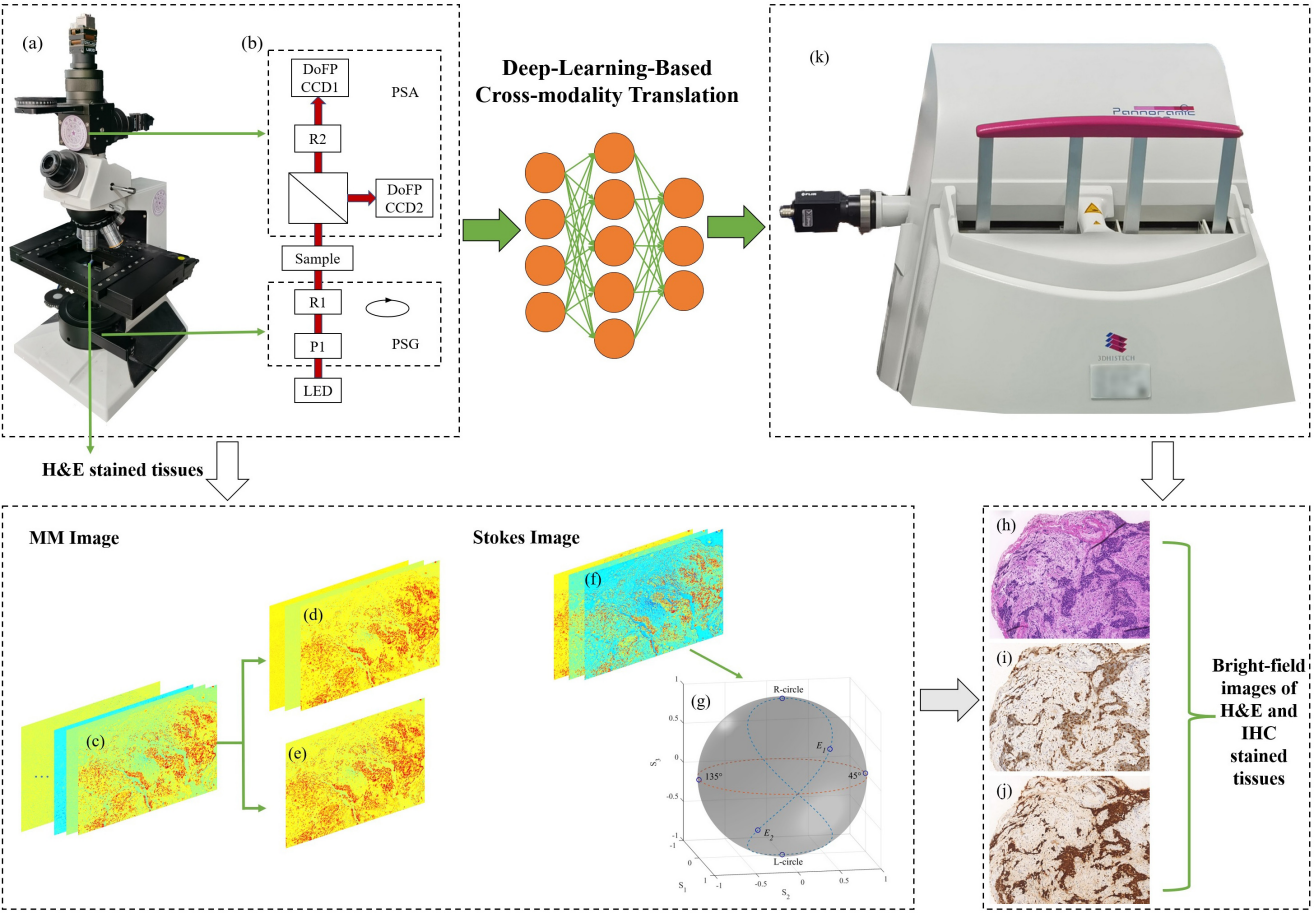
The workflow of cross-modality translation. (a) Photograph of DoFPs-MMM. (b) Configuration of DoFPs-MMM. (c) 16 MM element images. (d) Decomposed top three channels by PCA. (e) Decomposed top one channel by PCA. (f) Stokes images obtained with a single shot. (g) Six incident polarization states selected on Poincaré sphere. (h) Bright-field images on H&E stained tissues. (i), (j) Different types of bright-field images on IHC stained tissues. (k) WSI system.

For image collections, we used the dual DoFP polarimeter-based full MM microscopy (DoFPs-MMM).^25^ As shown in Figs. 1(a) and 1(b), the light from the LED (3 W, 632 nm, and Δλ=20  nm) is modulated by the polarization state generator (PSG) and then passes through the sample, whose scattered light enters the objective lens and is finally received by the polarization state analyzer (PSA). The PSG contains a fixed-angle linear polarizer P1 and a rotating zero-level quarter-wave plate R1. Before putting in use, the PSA needs to be calibrated first, after which the instrument matrix $A_{\mathrm{PSA}}$ can be calculated. Then we can obtain the complete Stokes vector of light scattered from the sample according to $S_{\text{out}}=A_{\mathrm{PSA}}^{-1}I$, where I denotes a column vector containing eight intensity images captured by the two DoFP polarimeters of the polarization directions corresponding to 0 deg, 45 deg, 90 deg, and 135 deg. We can gain the Stokes vector $S_{\text{out}}$ containing four components from a single shot, which can be expressed as $S={\left(S_{0},S_{1},S_{2},S_{3}\right)}^{⊺}$, where $S_{0}$ denotes the intensity images and $S_{1}-S_{3}$ are the components related to polarization. To achieve MM imaging, PSG is needed to generate four incident polarization states $\left[S_{\text{in}}\right]$ by rotating R1 to four preset angles and record the corresponding four Stokes vectors $S_{\text{out}}$ obtained from four exposures of the DoFP polarimeters. The full MM can be calculated by $M=[S_{\text{out}}]{\left[S_{\text{in}}\right]}^{-1}$. DoFPs-MMM has a faster acquisition speed and measurement accuracy^25^ compared to the dual rotating retarders-based MMM (DRR-MMM).^41^ It is faster using snapshot Stokes imaging than MM imaging in the DOFPs-MMM.

### 2.2 Sample Preparation and Image Acquisition

To validate the method on different tissues, we collected liver samples from 50 patients, breast samples from 22 patients and lung samples from 9 patients. Each patient represents a subject. The polarimetric properties of these samples have been analyzed in previous studies.^9^^,^^10^^,^^42^^–^^45^ Liver samples were prepared by Fujian Medical University Cancer Hospital and Mengchao Hepatobiliary Hospital of Fujian Medical University. Breast and lung tissue samples were obtained from the University of Chinese Academy of Sciences Shenzhen Hospital. All tissues were cut into sections of uniform 4  μm thickness. To observe different types of computational staining, all liver and breast tissue slices were stained with H&E, while different adjacent tissue slices of the lung samples were stained separately with H&E and different types of IHC for comparison. The bright-field RGB images were acquired by a whole slide image system (WSI). Breast and liver tissue H&E staining slices were captured using MMM and WSI system, respectively. Lung tissue H&E staining slices were captured using MMM and IHC staining slices were captured using WSI system. In this case, MM images and Stokes images could be separately obtained using MMM. We imaged part of the samples with a 4× objective lens and the others with a 20× objective lens. All works were approved by the Ethics Committee of these three hospitals. The number of samples are listed in the “image acquisition” column of Table 1. Ki-67 and thyroid transcription factor-1 (TTF-1) are two types of IHC staining.

**Table 1:** The details of data collection and dataset division. PM, polarimetric microscopy; BFM, bright-field microscopy.

	PM → BFM	Image acquisition		Training		Testing	
		subjects	regions	subjects	patches	subjects	patches
Liver tissue	HE → HE (paired)	50	183	38	1309	12	520
Breast tissue	HE → HE (paired)	22	340	15	1303	7	408
Lung tissue	HE → Ki−67 (unpaired)	9	40	7	580	2	220
	HE → TTF−1 (unpaired)	9	40	7	640	2	160

### 2.3 Data Pre-Processing

MM contains all polarimetric properties about the samples. Considering the comprehensiveness of the information, we utilized all elements in MM. There is a significant correlation among the 16 elements in the MM, leading to unnecessary data duplication (redundancy).^46^ The correlations between different MM elements are determined by the sample’s polarization properties, but this correlation still relies on the data distribution from a statistical perspective. We used principal component analysis (PCA) to extract most of the information related to polarization properties from the initial MM. PCA has been widely used in multivariate image analysis for dimensionality reduction, data compression, pattern recognition, visualization,^47^ etc. In this part, it decomposed 16 MM element images into a linear combination of few uncorrelated basis functions. We utilized the top one channel (PCA1) or three channels (PCA3), which explain most of the variance within the dataset. An overview of the MM imaging and data preprocessing procedure is given in Figs. 1(c)–1(e).

The Stokes images were obtained by a single shot using DOFPs-MMM. $S_{1}$, $S_{2}$, and $S_{3}$ images, all being normalized by the intensity image $S_{0}$, could be transformed as three channels of an RGB image. The incident state of polarization (SOP) can determine the outgoing Stokes vector. As shown in Fig. 1(g), to demonstrate our method is incident SOP-independent, all forms of complete polarization states (linear, circular, and elliptical polarization) are included, with each form of SOP being paired orthogonally. We selected two circular polarization (right-hand circle and left-hand circle) and two elliptical polarization ($E_{1}$ and $E_{2}$) on the traces of polarization states of the Poincaré sphere generated by continuously rotating R1 180 deg in PSG,^25^ where $E_{1}={\left(1.0000.7500.4330.500\right)}^{⊺}$ and $E_{2}={\left(1.0000.750-0.433-0.500\right)}^{⊺}$. For a more general consideration, we deviate from the aforementioned traces and selected two linear polarizations (45 deg and 135 deg) on the equator of the Poincaré sphere.

The decomposed MM images and Stokes images were used as input, respectively, and the bright-field images were used as ground truth. When performing cross-modality translation on images of H&E stained slices, paired images were required to verify the transformation performance of the model. A speeded-up robust feature algorithm-based feature point detection image registration technique^48^ was used to build the dataset, ensuring that the polarimetric images and bright-field images were matched exactly at the pixel level. All of the images were scaled and cropped into 256×256  pixels patches.

### 2.4 Dataset and Implementation

Before training a cross-modality translation deep learning model, a dataset containing a large amount of polarimetric images and bright-field images needs to be built first. Paired images were used for breast tissues and liver tissues, while unpaired images were used for lung tissues. The patches in the training set and the patches in the test set did not overlap with each other and were from different patients. The division of the dataset is given in the “training” and “testing” columns of Table 1. Both the training and test sets contain images with 4× and 20× objective lens magnification.

The experiments were carried out on a desktop computer, of which the operating system is Ubuntu 18.04 with kernel 5.4.0. We used PyTorch 1.12.1 and Python 3.8.5 to train models and implemented them on two NVIDIA Geforce RTX 2080Ti cards. Each model was trained with a batch size of 4 for 100 epochs, where the first 50 epochs were with the initial learning rate and the last 50 epochs linearly decayed learning rate to zero.

## 3 Translation Model

**Fig. 2:**
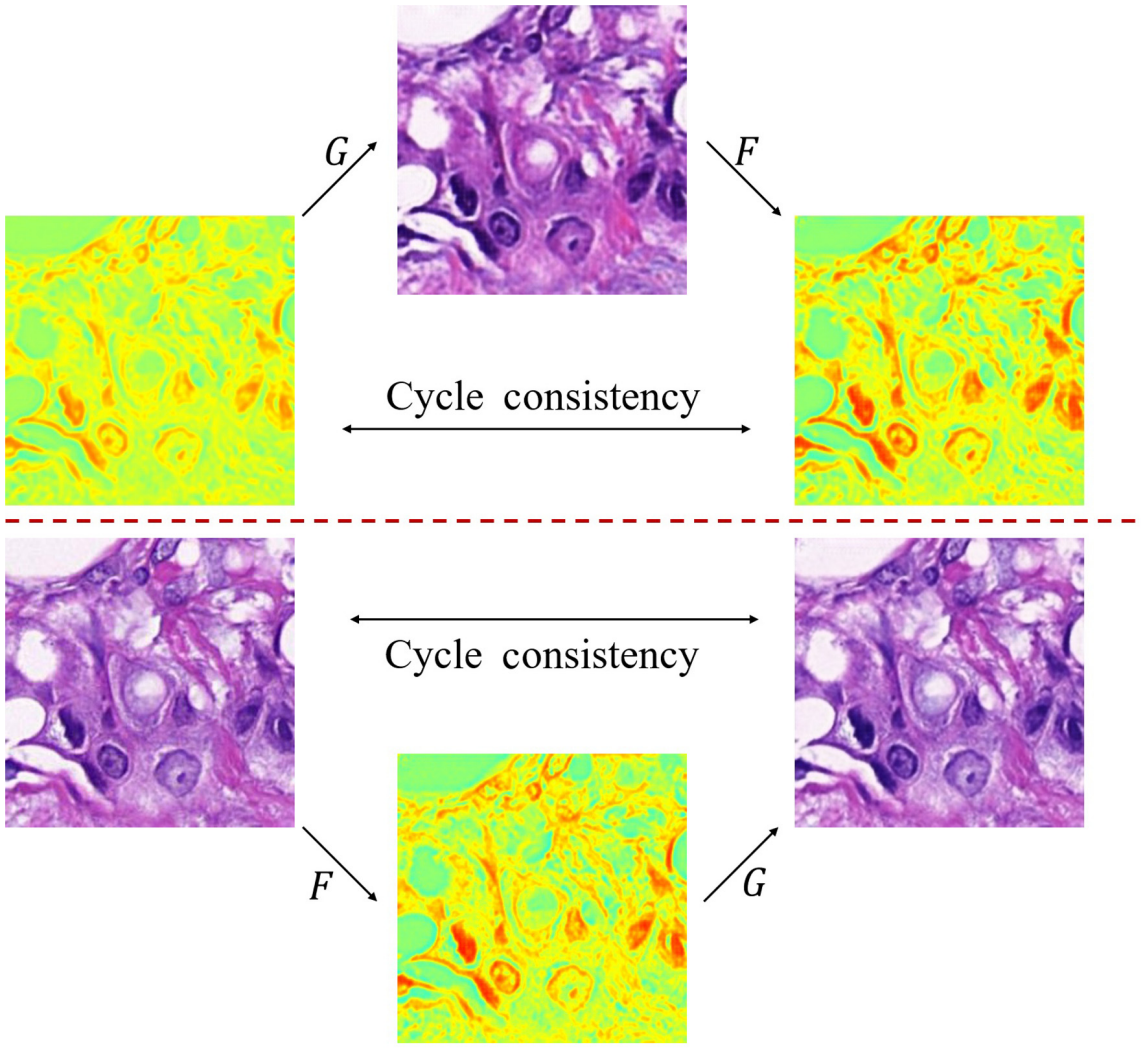
Graphical overview of CycleGAN model during the training process. The cycle transformation from polarimetric image to bright-field image x→G(x)→F(G(x))≈x is shown on the top part. The cycle transformation from bright-field image to polarimetric image y→F(y)→G(F(y))≈y is shown on the bottom part.

Aiming at the task of cross-modality translation from polarimetric images (including MM images and Stokes images) to bright-field images, we used the CycleGAN model,^40^ which includes two generators that can convert between X and Y with each other, where G is the mapping X→Y and F is the mapping Y→X. The schematic diagram of the translation is shown in Fig. 2.

CycleGAN contains both generators and discriminators. The generator G can take a polarimetric image x←X by $y^{\prime }=G(x)$ and generate a bright-field image that is as similar as possible to the real image. The discriminator $D_{Y}$ learns to distinguish whether the bright-field image is real (labeled as 1) or generated (labeled as 0). This is the original adversarial loss, which can be expressed as

$$L_{\mathrm{GAN}}(G,D_{Y},X,Y)=E_{y∼Y}[\log\;D_{Y}(y)]+E_{x∼X}[\log(1-D_{Y}(G(x)))].$$ (1)

G tries to minimize the goal to counter $D_{Y}$’s attempt to maximize it. To strengthen the constraints of the mapping relationship, it is coupled with F that inversely maps to a polarimetric image to ensure that F(G(x))≈x. $L_{1}$ penalty is introduced as the reconstruction error, as shown in Eq. (2), which is called cycle consistency loss:

$$L_{\mathrm{cyc}}(G,F,X)=E_{x∼X}[{\left\|F(G(x)-x)\right\|}_{1}].$$ (2)

Similarly, the translation of a bright-field image to a polarimetric image contains both the adversarial loss of F and $D_{X}$ and the cycle consistency loss between Y and G(F(y)). The overall objective is

$$G^{*},F^{*}=\arg\;\underset{G,F}{\min}\;\underset{D_{x},D_{y}}{\max}\;L_{\mathrm{GAN}}(G,D_{y},X,Y)+L_{\mathrm{GAN}}(F,D_{x},Y,X)\;+\lambda (L_{\mathrm{cyc}}(G,F,X)+L_{\mathrm{cyc}}(F,G,Y)).$$ (3)

**Fig. 3:**
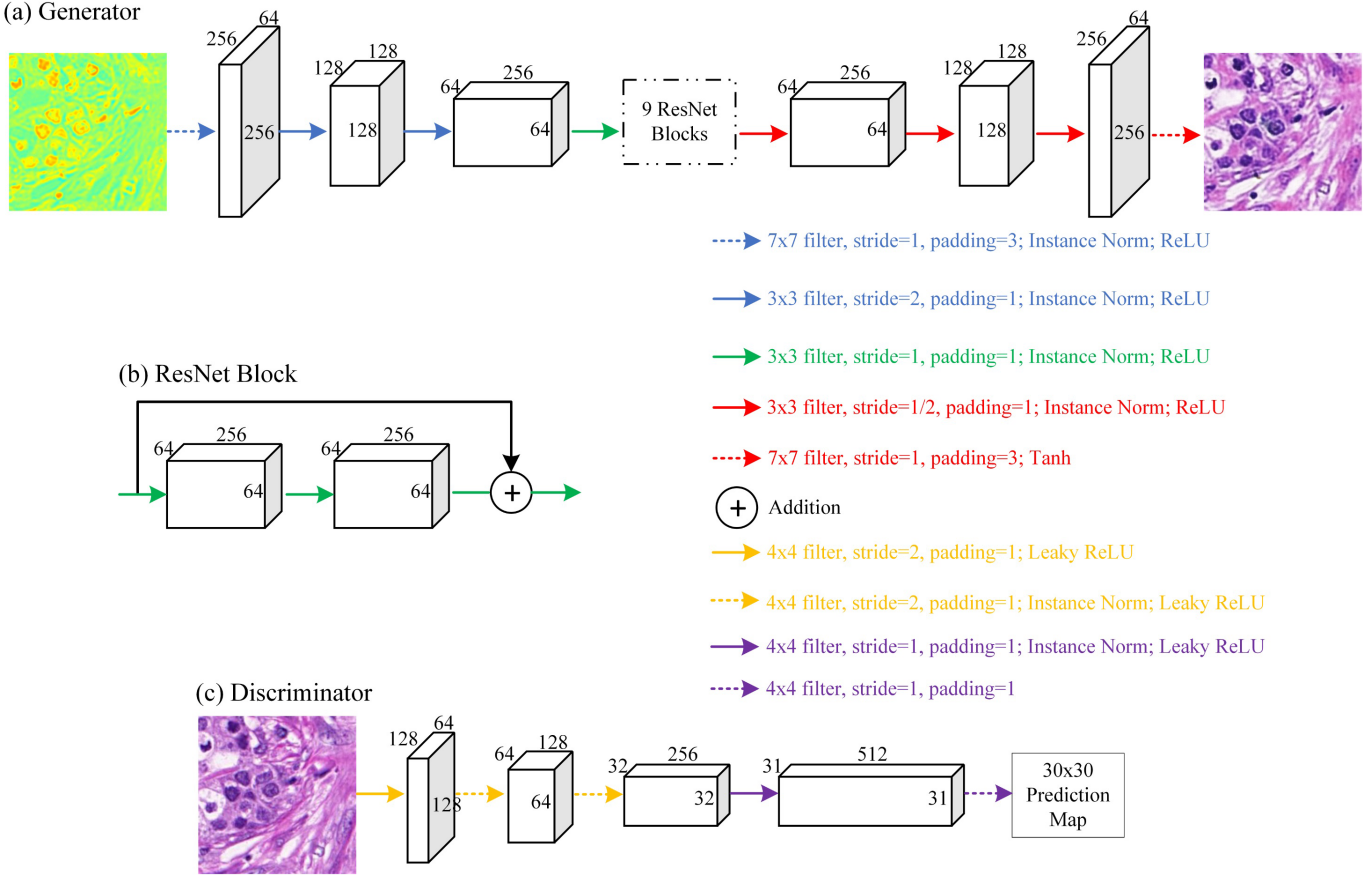
(a)–(c) Cross-modality translation model architecture.

The architecture of the cross-modality translation model adopts ResNet-based on a generator with nine residual blocks, which also performs downsampling and upsampling operations, as shown in Fig. 3(a). The 256×256×1 (PCA1) or 256×256×3 (PCA3 or Stokes image) polarimetric image is input to the generator and both downsampling and upsampling consist of three steps. Each step of downsampling contains a convolutional layer, while each step of upsampling contains a deconvolutional layer, and both are followed by an instance normalization process and a rectified linear unit (ReLU). A hyperbolic tangent function (tanh) is included after the last upsampling feature map of size 256×256×64 to output the 256×256×3 bright-field image. The residual block adds a shortcut connection of the feedforward neural network (skipping one or more layers of connections) to achieve identity mapping and connects its outputs to the outputs of the stacked layers.^49^ A block is shown in Fig. 3(b). Figure 3(c) shows the structure of the discriminator, which uses a 70×70 PatchGAN.^50^ In this process, PatchGAN tries to identify whether each 70×70 patch is real or fake. The discriminator needs to input a 256×256×3  pixel patch and output a 30×30 prediction map.

Polarimetric images (including MM and Stokes images) represent the polarization properties of a sample, which are closely associated with its microscopic structures. The contrast of bright-field microscopy images is caused by the light attenuation in different regions of microstructures. Therefore, there is a strong correlation between the input data (polarimetric images) and the generated data (bright-field images). Compared to bright-field microscopy, polarization-based measurement methods have the capability to detect high-dimensional information and specific structural characteristics. The proposed cross-modality translation model is able to extract key features relevant to microscopic structures from the input high-dimensional polarimetric data and then gradually reconstruct the bright-field image based on them.

## 4 Results

### 4.1 Results on H&E Stained Tissues

**Fig. 4:**
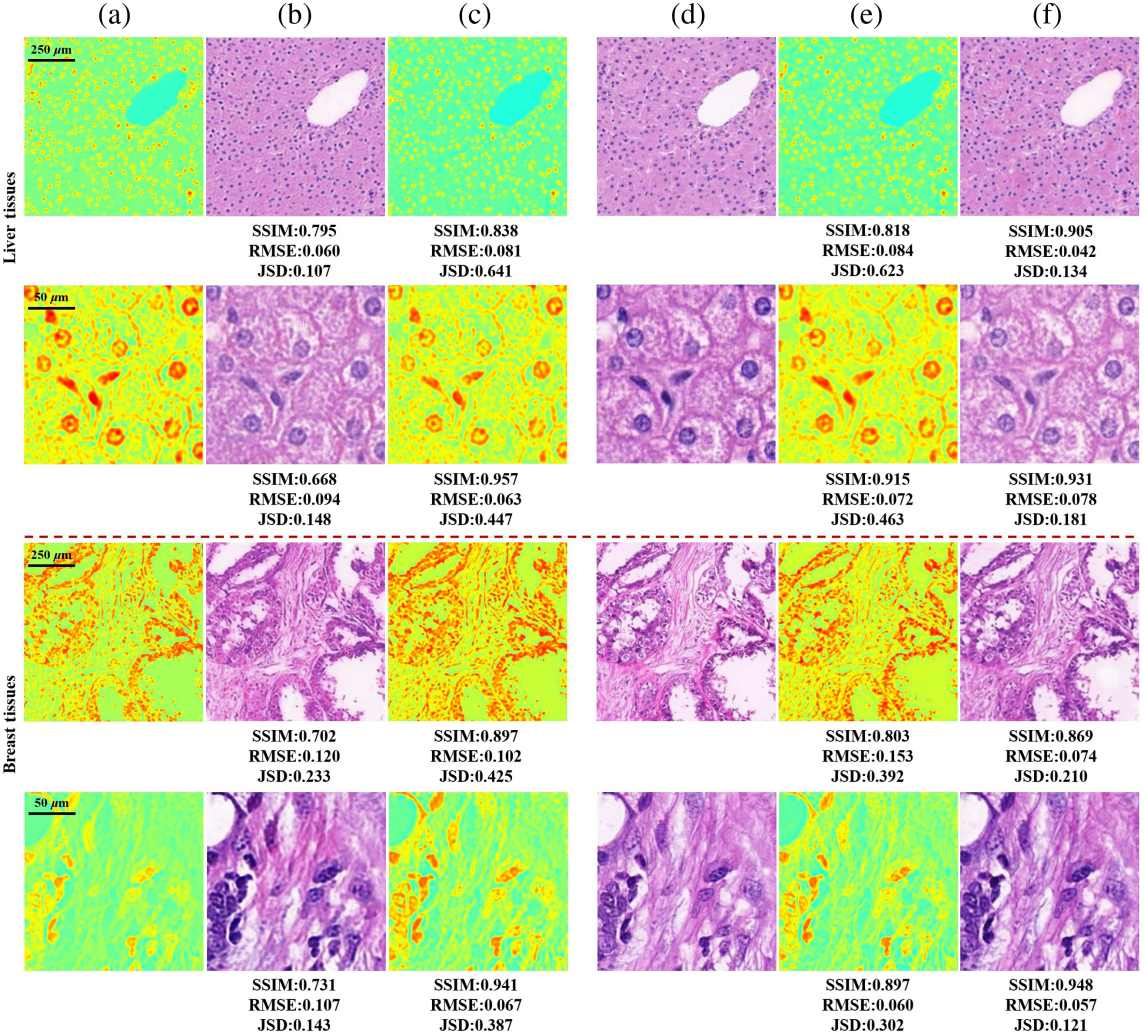
Cycle results of cross-modality translation on liver and breast tissues. The first and second rows of each tissue show the results on the sample imaged by 4× and 20× objective lenses, respectively. (a) 3 channels Stokes images x, illuminated with 45 deg linear polarization light. (b) Generated bright-field images G(x). (c) Reconstructed Stokes images F(G(x)). (d) Bright-field images y. (e) Generated Stokes images F(y). (f) Reconstructed bright-field images G(F(y)). (b), (f) The quantitative differences with panel (d). (c), (e) The quantitative differences with panel (a).

We first applied the proposed model on the liver and breast sample images acquired by illuminating with single 45 deg linear incident polarization state. It could validate the feasibility of CycleGAN on the task of cross-modality translation from Stokes images to bright-field images by paired images of the two samples. An input source image x from domain X was converted to a target image G(x) and then was cyclically converted back to F(G(x)) that was close to x. A Similar conversion was completed from y to G(F(y)). We trained images mixing 4× and 20× objective lens magnification and tested the model on both scales together. Figure 4 shows the transformative powers of both G and F. It showed that the reconstructed images F(G(x)) and G(F(y)) matched closely with the real images x and y from X and Y domains. For paired images, the translated images G(x) and F(y) were very similar to y and x, respectively. This illustrates that for a model being well trained on multi-scale Stokes images, it can get excellent transformation performance on corresponding scale images in the prediction. The insensitivity to resolution reduces model training for different scale images and improves robustness of the deep model.

Then we separately selected the Stokes images obtained from six SOPs shown in Fig. 1(g) as the input of the deep learning-based model. The quantitative comparison results of bright-field images on liver and breast tissues are given in the “Stokes images” row of Table 2. We adopted structural similarity (SSIM) index,^51^ root mean square error (RMSE),^52^ Jensen–Shannon divergence (JSD)^53^ and Earth mover’s distance (EMD)^54^^,^^55^ on the testing data. SSIM and RMSE measure the difference between two images at the image and pixel level, respectively. JSD and EMD are used to measure the distance between two distributions. Results with different SOPs are close to each other, which imply the model is not sensitive to the incident polarization state as far as it contains all the linear and circular polarization components, i.e., $S_{1}$, $S_{2}$, and $S_{3}$. It can reduce the complexity of PSG and improve the robustness of the system.

**Table 2:** Quantitative comparison of cross-modality translation from MM and Stokes images to bright-field images of liver and breast tissues. Bold value represents the maximum value of each column.

		Liver tissue				Breast tissue			
		SSIM	RMSE	JSD	EMD	SSIM	RMSE	JSD	EMD
MM images	PCA1	0.722	0.097	**0.174**	9.216	0.744	**0.119**	**0.187**	**7.990**
	PCA3	0.690	0.104	0.192	9.620	0.706	0.142	0.202	10.637
Stokes images	45 deg	0.694	0.099	0.181	8.935	0.727	0.130	0.200	9.761
	135 deg	0.710	0.104	0.203	11.515	0.742	0.126	0.191	9.296
	R-circle	**0.732**	0.099	0.178	9.353	0.742	0.139	0.202	11.955
	L-circle	0.713	0.101	0.186	9.077	0.752	0.134	0.226	11.711
	$E_{1}$	0.706	0.102	0.209	11.093	**0.760**	0.126	0.202	9.796
	$E_{2}$	0.718	**0.093**	0.178	**8.467**	0.755	0.124	0.192	9.460

**Fig. 5:**
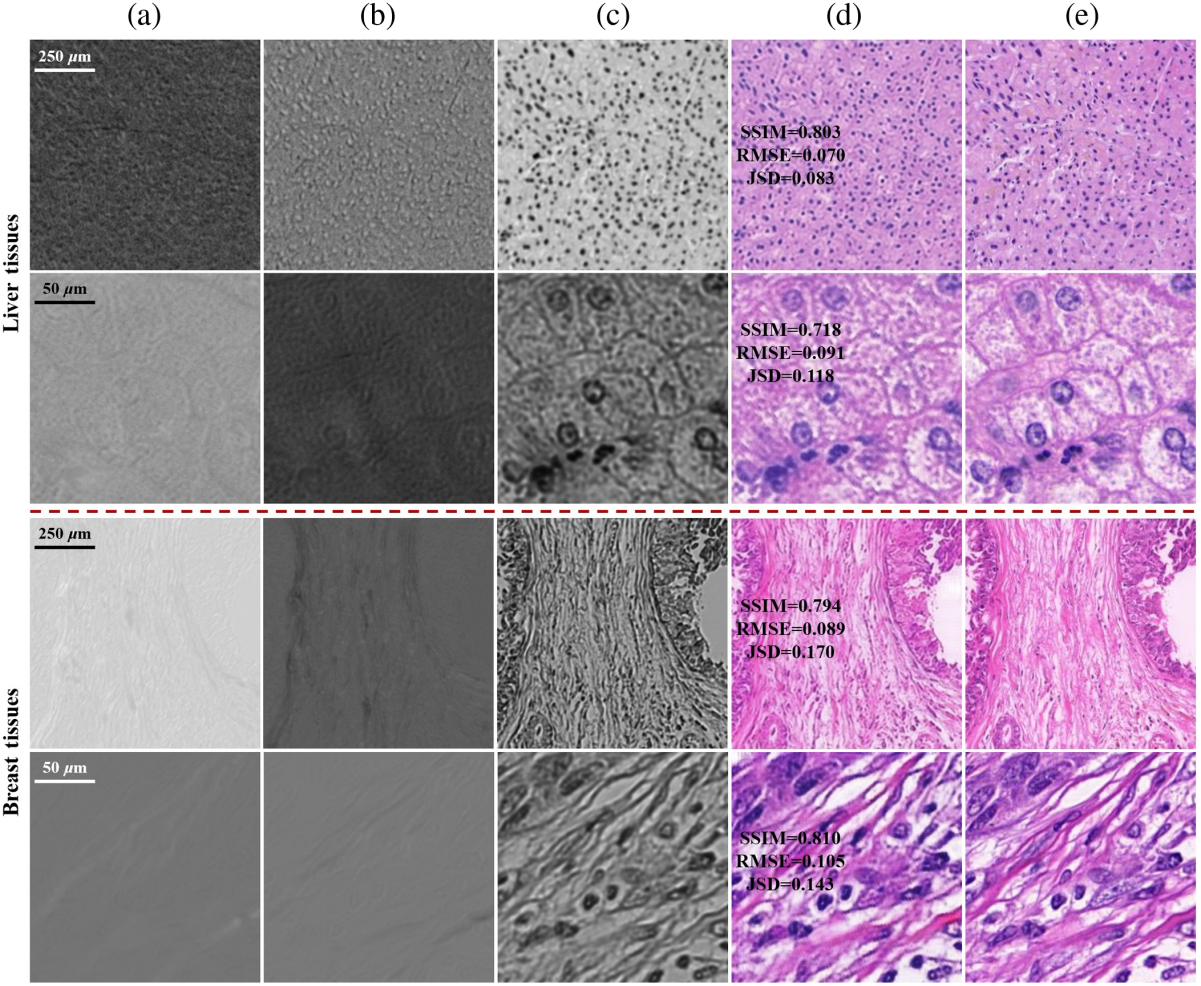
Generated results of bright-field images from Stokes images captured by 4× and 20× objectives lenses are shown in the first and second rows on liver and breast tissues, respectively. (a)–(c) Normalized images of $S_{1}$, $S_{2}$, and $S_{3}$ in $S_{\text{out}}$, respectively, illuminated with right-hand circle polarization light. (d) Generated bright-field images. (e) Ground truth captured by bright-field microscopy. (d) The quantitative differences with panel (e).

Figure 5 gives the results generated from single Stokes component with circular SOPs of incident light. The generated images accurately predict the spatial location and contour of the tissue, as well as the major features, such as nucleus morphology and fiber distribution, are comparable to ground truth. The color distribution is effectively restored, which conforms the human visual system. In the testing phase, the generator could convert a 256×256 Stokes image to the corresponding bright-field image by forward propagation within 0.1 s, which shows nearly real-time performance. The time to obtain a bright-field image of the corresponding field of view (FOV) in the MMM using the “bright-field snapshot MM microscopy” method depends on the frame rate of the CCD (0.1 s) and the translation time. The translation time can be reduced with a more powerful computer.

**Fig. 6:**
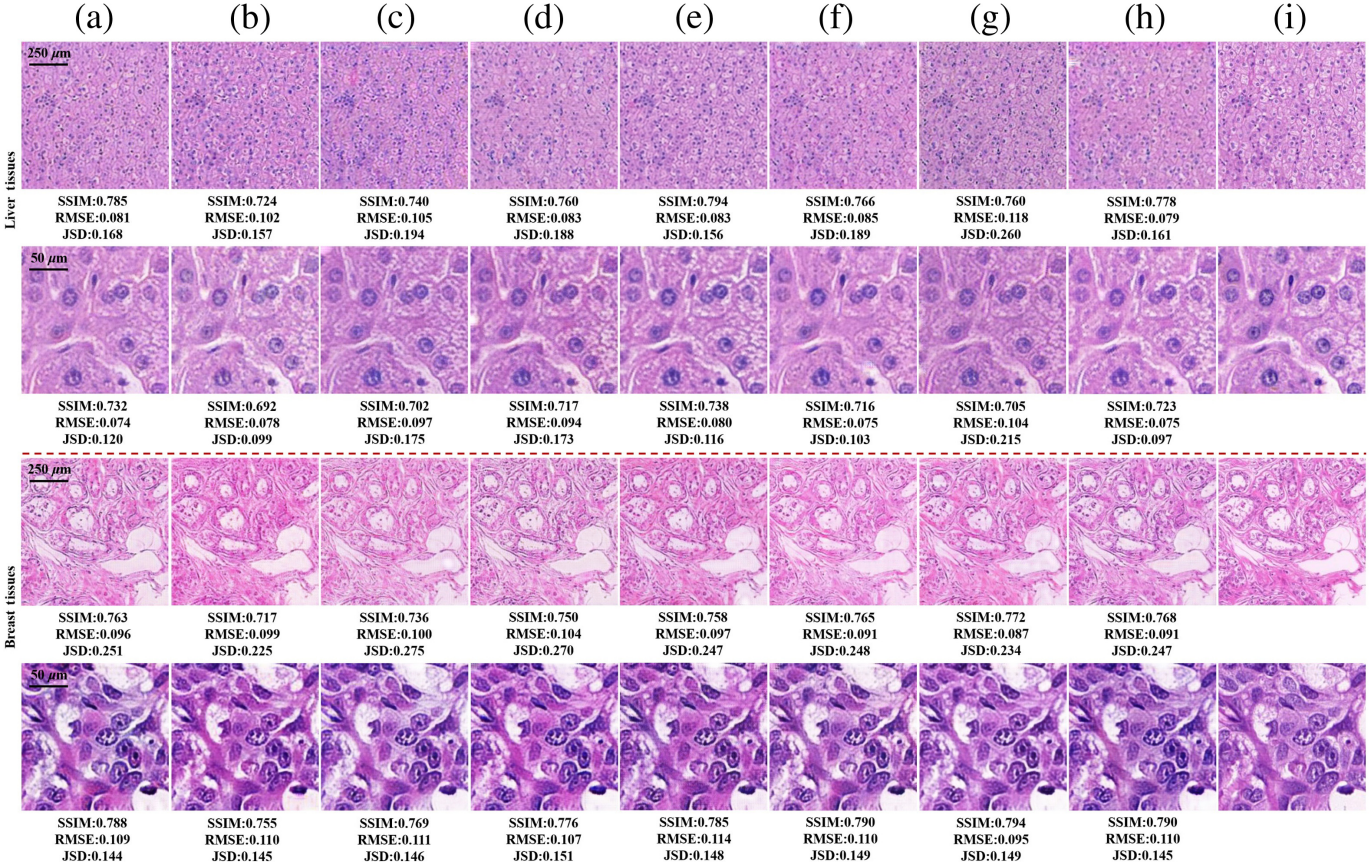
Comparison results of bright-field images on MM images and Stokes images of liver and breast tissues. The first and second rows of the two tissues are the transformation results at 4× and 20× objective lens magnification, respectively. (a), (b) Generated images of PCA1 (MM images of top one PCA channel) and PCA3 (MM images of top three PCA channels). (c)–(h) Generated images illuminated with 45 deg and 135 deg linear polarization light, right-hand and left-hand circle polarization light, and right-hand and left-hand elliptical polarization light $E_{1}$ and $E_{2}$ respectively in order. (a)–(h) The quantitative differences with panel (i), respectively.

We also translated the MM images to bright-field images for comparison with the generated results of Stokes images. We fed images processed by PCA with the first channel and the first three channels into our model. The output bright-field images were evaluated quantitatively, as given in Table 2 “MM images” row, and the visual comparison results are illustrated in Fig. 6. It can be seen that the model predicts the presence of different histological structures and cell types. In the images generated at low resolution, the details of structure are not particularly clear, but the tissue’s overall distribution can be discerned. In the high-resolution images, cell nucleus location, stroma, and cytoplasmic detail can be seen in nearly all images. All output images are close to each other and very well matching with the bright-field images. We can conclude that the model achieved comparable performance when inputting Stokes images than MM images. A Stokes image (0.1 s) is acquired more faster than an MM image (9 s) and it will eliminate errors introduced by sample motivation and system instability in exposures and the image co-registration process.^26^ Bright-field snapshot MM microscopy can improve the performance of cross-modality translation from MM microscopy to bright-field microscopy.

We need to train the models separately for liver and breast tissues, which requires a large amount of data and consumes computational resources. Transfer learning enables applying knowledge or patterns learned on a domain to a different but related domain.^56^ It can improve the learning performance of the target task based on migrating knowledge structures in the relevant domain. The morphological characteristics of different types of tissues are similar under MM and bright-field microscopy. We used the knowledge learned from liver tissue as the initialization of training the breast tissue model, which can reduce the convergence time and improve the generality.

**Fig. 7:**
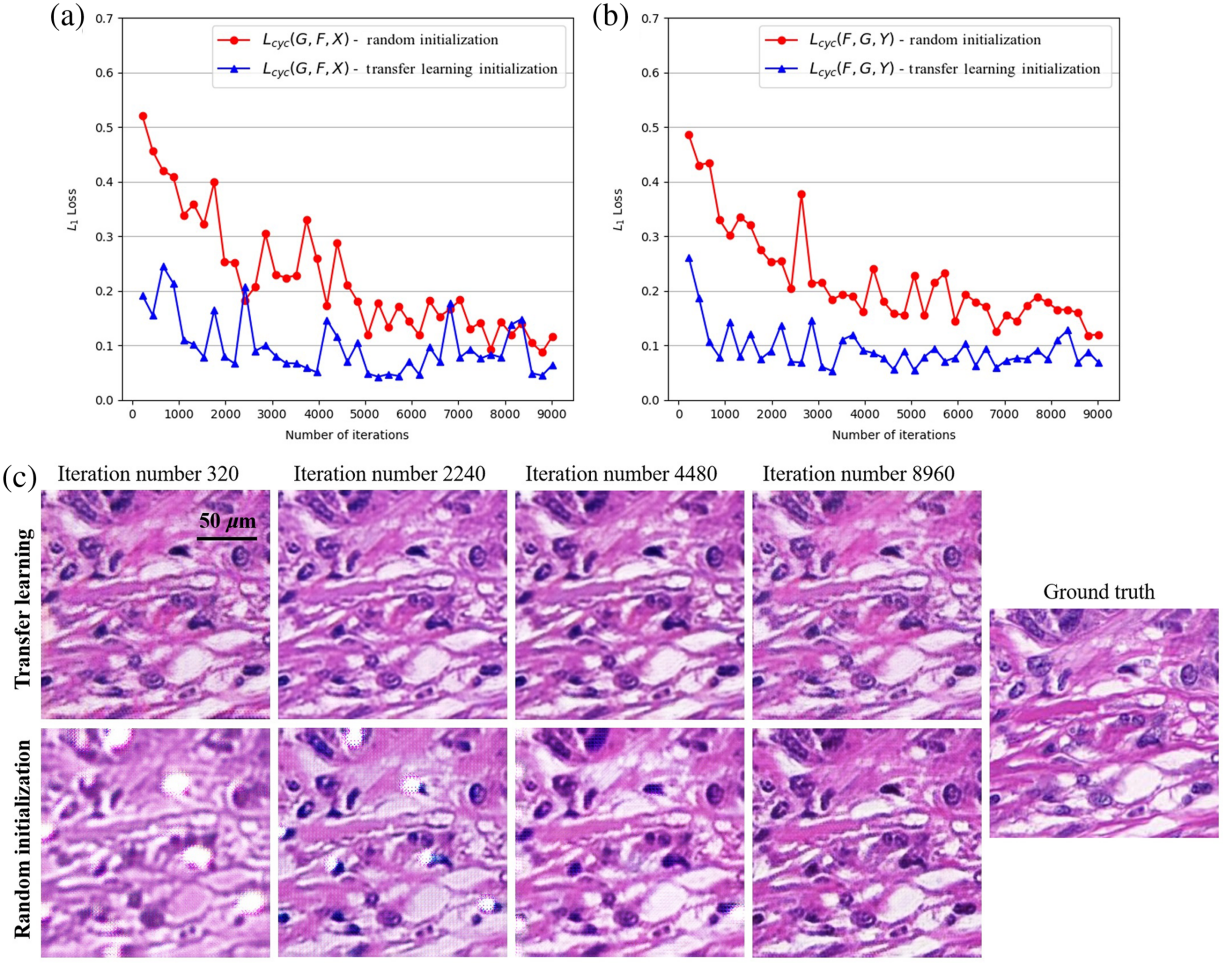
Consistent losses and generation results with increasing number of iterations. (a) Cycle consistency loss ($L_{1}$) values of forward translation $E_{x∼X}{\left[\|F(G(x))-x\|\right]}_{1}$ on breast tissues with random and transfer learning initialization, respectively. (b) Cycle consistency loss ($L_{1}$) values of backward translation $E_{y∼Y}{\left[\|G(F(y))-y\|\right]}_{1}$ on breast tissues with random and transfer learning initialization, respectively. (c) Generated images of breast tissues with transfer learning and random initialization.

The history of training losses can reveal the performance of the deep model during the training phase. Our goal is to generate bright-field images that match the target as closely as possible. The pixel-wise similarity loss ($L_{1}$) can quantitatively indicate the similarity. Figures 7(a) and 7(b) show the comparison of consistent losses for both of the two generators G and F, respectively. As we can see, all training procedures can lead to stable results and the model initialized with weights and biases learned from liver tissue converges faster than random initialization. Figure 7(c) visualizes the bright-field images generated at different iterations of transferring and not transferring on the breast tissue, to further demonstrate the impact of transfer learning.

### 4.2 Results on IHC Stained Tissues

Furthermore, we extended the network to two IHC staining types Ki-67 and TTF-1, which cannot access paired images. Ki-67 is a marker of cell proliferation and stains the nuclei. It is used for prognosis of relative responsiveness, resistance to chemotherapy or endocrine therapy, and as a biomarker of treatment efficacy (high percentage reflects a worse prognosis). TTF-1 is a nuclei marker with preferential expression in thyroid, lung, and brain structures of diencephalic origin. It is frequently used in the search for the primary origin of metastatic endocrine tumors.^57^

**Fig. 8:**
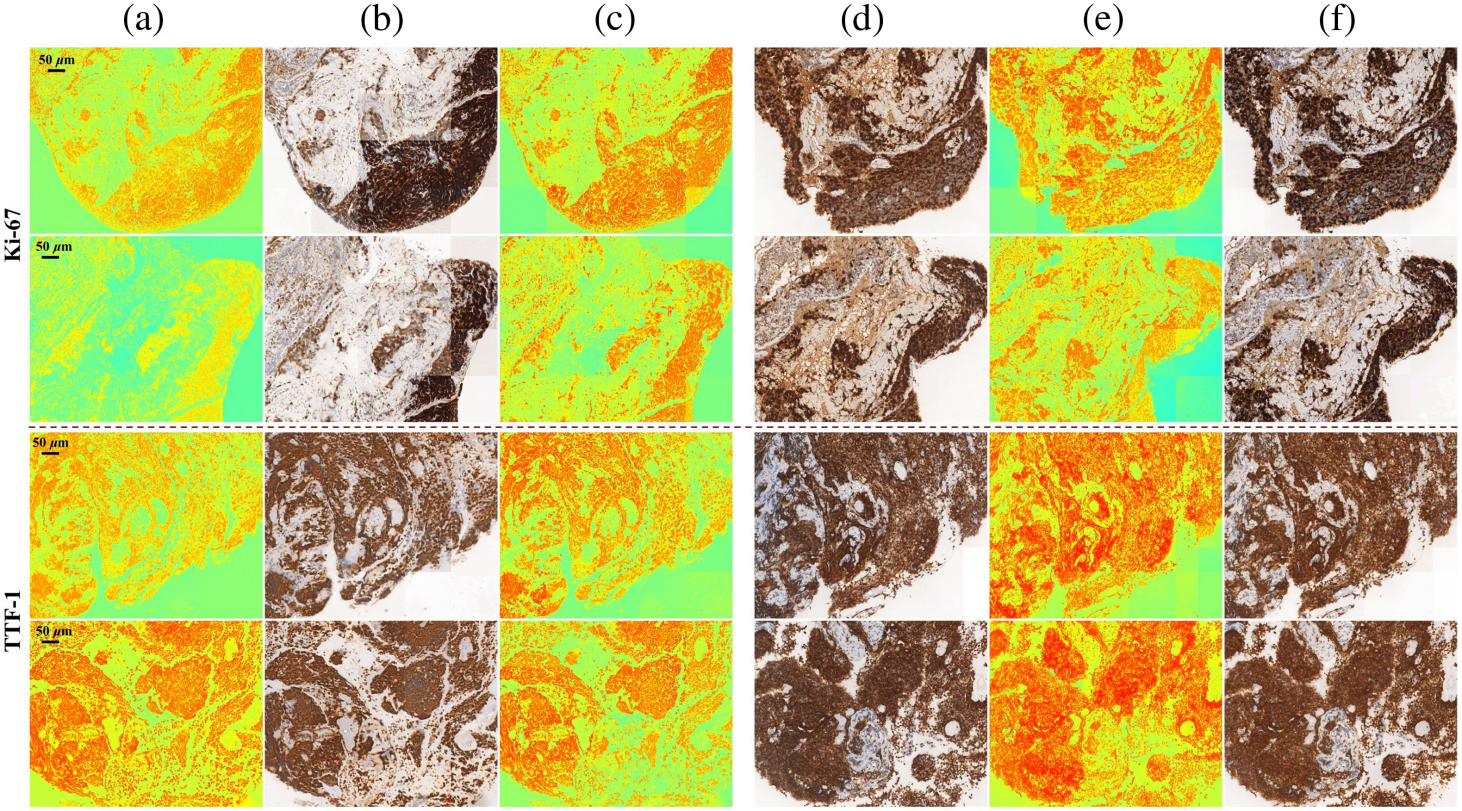
Stitched experimental cycle results of computational Ki-67 and TTF-1. (a) Input three channels Stokes images x, illuminated with 45 deg linear polarization light. (b) Generated bright-field IHC stained images G(x). (c) Reconstructed Stokes images F(G(x)). (d) Adjacent slice bright-field IHC stained images y. (e) Generated Stokes images F(y). (f) Reconstructed bright-field IHC stained images G(F(y)).

The results of similarity evaluation are listed in Table 3. Since there are no paired images, we used the error between input Stokes images x and reconstructed Stokes images F(G(x)) for evaluation. Figure 8 shows stitched whole images for these two types of computational staining. In this part, the ground truth is the adjacent slice. There is always some degree of inter-slide variation between the matched slides, but they share similar semantic and structural features. Examination by an experienced pathologist indicated that the generated images are capable of predicting the presence and location of markers, presenting the overall pathological information of the sample.

**Table 3:** Quantitative comparison of computational staining based on cross-modality translation between x and F(G(x)) of lung tissues. Bold value represents the maximum value of each column.

		Ki-67				TTF-1			
		SSIM	RMSE	JSD	EMD	SSIM	RMSE	JSD	EMD
MM images	PCA1	0.926	0.082	0.241	8.188	**0.926**	0.098	0.257	8.195
	PCA3	0.923	0.080	0.233	7.729	0.903	0.100	0.274	7.844
Stokes images	45°	**0.929**	0.078	0.237	7.520	0.915	**0.087**	**0.246**	**6.698**
	135°	0.923	**0.075**	**0.229**	**6.870**	0.904	0.101	0.277	8.384
	R-circle	0.917	0.083	0.248	7.914	0.917	0.092	0.267	7.360
	L-circle	0.911	0.081	0.237	7.322	0.925	0.092	0.261	7.519
	$E_{1}$	0.920	0.082	0.240	7.738	0.914	0.097	0.263	7.998
	$E_{2}$	0.908	0.083	0.239	7.693	0.914	0.091	0.253	7.194

## 5 Discussion and Conclusion

In this work, we presented a cross-modality translation method that can obtain bright-field image of different staining styles in snapshot Stokes imaging. It is not only time-, labor-, and cost-saving but also avoids errors caused by light intensity instability and image misregistration in the MMM. The application is based on CycleGAN without pixel-wise paired examples in the training. This can reduce the workload in the data preparation and is especially essential when the equivalent ground truth is difficult to acquire or even unavailable. We first used MM and bright-field microscopy to capture images in the same region of stained sections to demonstrate the performance of the deep learning model, and then used the two microscopic devices to take H&E stained sections and IHC stained adjacent sections respectively to achieve cross-modality translation with computational staining on Ki-67 and TTF-1 IHC staining styles. Using this approach, a DoFP polarimeter-based MMM can simultaneously acquire polarimetric images and bright-field images of multiple staining styles in the same FOV in a single shot. In the experiments, we trained and tested on a collection of stokes images at both 4× and 20× magnification with multiple SOPs on liver and breast tissues. The generated results demonstrated that it was resolution and SOP insensitivity to polarimetric images, which improves the robustness of the system for cross-modality translation. Transfer learning can accelerate the convergence process on new tasks based on the knowledge learned from a well-built one.

There are many other unexplored possibilities for cross-modality translation based on polarimetric images. It has been demonstrated that the wavelength of light has an effect on the polarimetric properties of the sample.^58^ Next, we will try to apply this model to learn relations and mappings between different wavelengths in MM polarimetry. In addition to brightfield microscopy, pathological analysis sometimes relies on other different imaging systems that have their own advantages. Polarimetric data contain high-dimensional information and are sensitive to microstructures in tissue, which makes it possible to discover relationships with other imaging systems, e.g., phase imaging^59^ and fluorescence.^60^ In addition, more powerful deep learning models, such as transformers,^61^ have recently been proposed. In future work, we will try to train the model to generate images of other imaging systems from polarimetric images. Furthermore, as pathologists usually require various staining reagents to provide additional contrast of different tissue components, this translation model can also be applied for other staining styles.

## Code, Data, and Materials Availability

Data underlying the results presented in this paper are not publicly available at this time but may be obtained from the authors upon reasonable request.
